# Radiographs and MRI of the Cervical Spine in Juvenile Idiopathic Arthritis: A Cross-Sectional Retrospective Study

**DOI:** 10.3390/jcm10245798

**Published:** 2021-12-11

**Authors:** Mateusz Kotecki, Piotr Gietka, Magdalena Posadzy, Iwona Sudoł-Szopińska

**Affiliations:** 1 Department of Radiology, National Institute of Geriatrics, Rheumatology and Rehabilitation, 02-637 Warsaw, Poland; sudolszopinska@gmail.com; 2 Department of Pediatric Rheumatology, National Institute of Geriatrics, Rheumatology and Rehabilitation, 02-637 Warsaw, Poland; malgieta1@gmail.com; 3 Individual Private Practice Magdalena Posadzy, 61-426 Poznan, Poland; magdalenaposadzy@gmail.com

**Keywords:** cervical spine, juvenile idiopathic arthritis, atlanto-axial subluxation, radiography, magnetic resonance imaging

## Abstract

Background: Juvenile idiopathic arthritis (JIA) is the most common rheumatic disease among children. In some patients, cervical spine arthritis remains a serious and chronic manifestation of JIA. The aim of this study was to assess the frequency of cervical spine lesions on radiographs and MRI in JIA patients with clinical signs of cervical spine involvement and to verify if with the addition of MRI, the use of radiographs could be abandoned. Methods: This retrospective study evaluated consecutive 34 children (25 girls; aged 6–18 years, median 15.5 years) with JIA and with clinical involvement of cervical spine. In each patient, both radiographs and MRI of the cervical spine were performed. Imaging findings were correlated with clinical and laboratory data. Results: The cervical spine was affected in 35% of patients. The most frequent lesions were subaxial subluxations (SAS; 24%), apophyseal joint ankylosis (9%), and C1/C2 joint lesions (9%). Anterior atlanto-axial subluxation (AAS) was diagnosed only by radiography, and most of the SAS were seen on radiography, whereas only a few on MRI. Reversely, C1/C2 soft tissue involvement were seen on MRI only. Cervical spine involvement was associated with raised ESR (*p* = 0.012) and CRP (*p* = 0.014). Conclusions: The cervical spine lesions are still frequent complication of JIA affecting up to 35% of JIA patients. Most of them develop serious complications, such as AAS and ankylosis. Despite advantages of MRI in terms of the imaging of the atlanto-axial region radiography shows superiority in diagnosis of AAS and SAS.

## 1. Introduction

Juvenile idiopathic arthritis (JIA) is a systemic inflammatory disease of poorly understood complex pathogenesis, influenced by genetic and environmental factors [1]. Recent classification divides JIA into seven subtypes: systemic onset JIA, oligoarthritis, rheumatoid factor (RF) positive polyarthritis, RF-negative polyarthritis, enthesitis-related arthritis (ERA), psoriatic arthritis (PsA), and undifferentiated arthritis (uA) [1]. It is the most commonly diseased spinal location that is affected by JIA in up 77% of individuals [2]. According to the American College of Rheumatology (ACR) and recent studies, the real prevalence of cervical spine involvement may, however, be higher due to the subclinical course of the disease [3,4]. For example Kjellberg et al. [3] in a study on 82 children with JIA showed that 35% of them developed at least one radiographic lesion in cervical spine. In a recent study performed on 40 children with JIA, only 20% of patients had clinical symptoms, while in 62.5% the lesions were identified by cervical spine MRI [5]. Moreover, the cervical spine involvement is considered a poor prognostic factor by ACR [4]. There is a lack of studies which focus both on radiography and MRI in children.

The spectrum of spinal lesions in JIA is similar to adults with rheumatoid arthritis (RA) except for a higher occurrence of ankylosis and vertebral bodies or discs hypoplasia in juveniles [6,7]. The most common abnormalities include apophyseal joint ankylosis, C1/C2 arthritis, and anterior atlanto-axial subluxations [6,7,8]. Compared to RA, erosions are found only occasionally [6,7,8,9]. Another peculiarity in JIA is vertebral bodies and intervertebral discs hypoplasia. Due to the natural joint laxity in children, the assessment of cervical instabilities and their differentiation with unstable pseudospondylolisthesis is challenging [7,9].

There is still no consensus whether to perform radiography or MRI for diagnosis of cervical spine involvement. Especially nowadays, when the prevalence of advanced cervical lesions should be less common in the era of biological treatment, the use of radiographs might be questioned. Radiography provides dynamic information on cervical spine alignment whereas it has low sensitivity for the detection of early inflammatory lesions. MRI enables imaging of complex atlanto-axial anatomy, inflammatory pannus, bone marrow edema and spinal cord lesions. Thus, the use of MRI may lead to early diagnosis of cervical spine arthritis. However, dynamic evaluation of the spine is not performed during MRI in majority of the centers. In radiography, another concern is radiation, whereas MRI is radiation-free. However, in younger children, to avoid motion artifacts sedation may be needed during MRI.

The majority of early lesions are reversible, but they may also progress to chronic irreversible abnormalities that will be seen on radiography, such as subluxations and ankylosis [10].

The aim of this study was to assess frequency of cervical spine lesions on radiographs and MRI in a cohort of JIA patients with clinical signs of cervical spine involvement and to verify if with the addition of MRI, the use of radiographs could be abandoned.

## 2. Materials and Methods

The local institutional review board has accepted the study protocol (no. KBT-3/2/2018). The study was performed respecting the ethical principles of the Declaration of Helsinki. 

**Figure 1:**
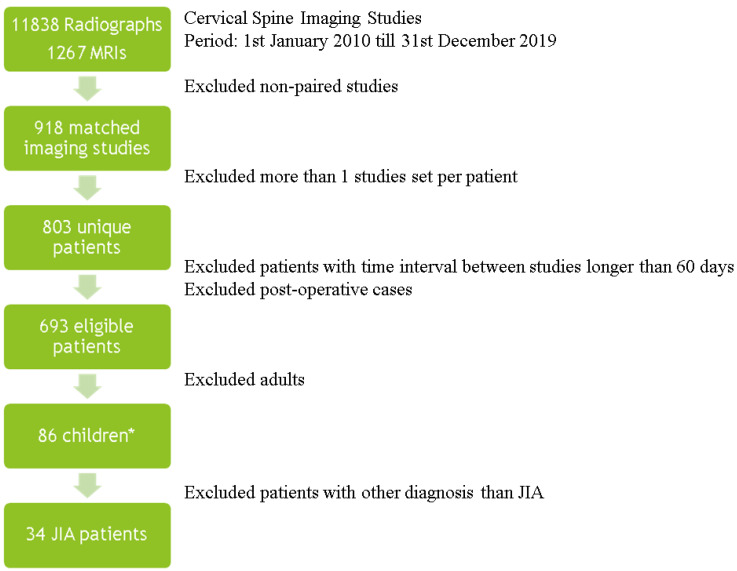
Summary of the inclusion process. *, 52 children with non-JIA diagnosis, after age and sex matching 38 patients, JIA—juvenile idiopathic arthritis, MRI- magnetic resonance imaging.

Lateral radiographs and MRI of the cervical spine performed in children clinically suspected of the cervical spine arthritis from 1 January 2010 to 31 December 2019 were assessed retrospectively. At single rheumatology center at this time period 11,838 radiographs and 1267 MRIs of the cervical spine both in adults and children were performed. From this group after pairing studies (at least one MRI and one radiograph per patient), excluding more than one study set per a patient, cases with time interval between studies exceeding 60 days, excluding adults, and finally excluding diagnoses other than JIA, the study group amounted to 34 patients. Fifty-two children had a diagnosis other than JIA, and age and sex matching served as the control group (38 patients). The Figure 1 show a general summary of an inclusion process. 

As the mentioned before, the study included 34 patients with JIA with a median age of 15.5 years (IQR 13.0–17.0) of which 25 were girls. Thirty-eight children with the diagnosis other than JIA were matched according to age and sex and served as a control group. No significant differences (*p* > 0.05) were found in the subject characteristics between both groups. This group included 3 children with juvenile systemic lupus erythematosus, 3 with juvenile scleroderma, 1 with borreliosis, and remaining 31 with numbness of upper extremity or cervical spine pain, without specific diagnosis and history of spine injury that could, e.g., indicate spinal cord injury without radiographic abnormality (SCIWORA). Comparison between JIA and non-JIA groups is shown on Table 1.

All patients were referred for imaging by rheumatologists at a reference center with clinical suspicion of cervical spine arthritis (pain, limited motion, or torticollis). All patients had been previously diagnosed with JIA. The symptoms suggesting cervical spine involvement were not present at initial diagnosis. Severe neurological symptoms were not observed.

All patients with JIA had both radiographs and MRI, whereas in the non-JIA group all patients had cervical spine radiographs and MRI was performed in 23 of them. The time interval between radiographs and MRI did not exceed 60 days. In the case that a patient had multiple cervical spine radiographs or MRI, the studies with the smallest time interval were included. 

Plain radiography in lateral neutral projection was supplemented in some patients by flexion and extension views. All 3 projections were performed in the erect patient, with left side against the upright detector. Following the neutral projection, functional views were performed, with patient’s neck in the gentle extension (chin up) or flexion (chin down) positions. The following lesions were evaluated on radiography: Demineralization (osteopenia, osteoporosis), cysts and erosions of the odontoid process, and atlanto-axial subluxations (AAS-anterior, or vertical a.k.a. basilar invagination/settling or cranial settling). On the subaxial level, subaxial subluxations (SAS), apophyseal joint ankylosis, vertebral and/or disc hypoplasia, and longitudinal ligament calcifications were reported [9]. 

**Figure 2:**
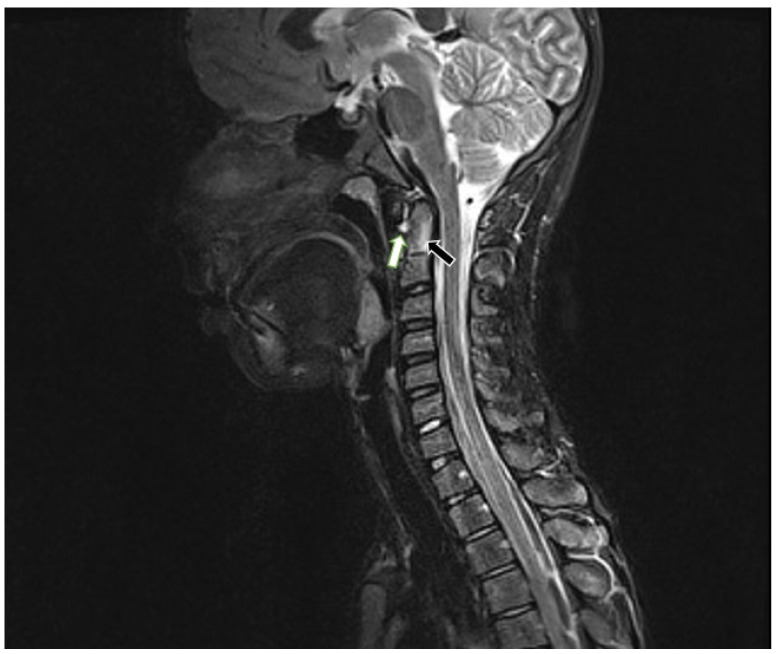
Sagittal MRI, TIRM T2w sequence in a 12-year-old boy diagnosed with enthesitis-related arthritis shows BME in the dens (black arrow) and atlantoaxial effusion (white arrow). MRI—magnetic resonance imaging, TIRM T2w-turbo inversion recovery magnitude T2 weighted, BME—bone marrow edema.

**Figure 3:**
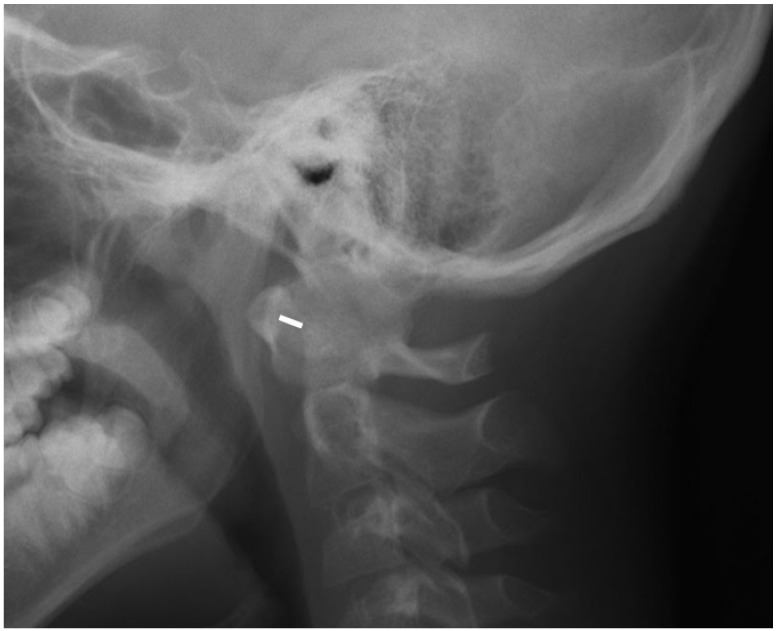
Lateral radiograph in neutral position in a 12-year-old boy (the same patient as in Figure 2) showing anterior atlantoaxial subluxation 6 mm (white line).

For the MRI examinations, a 1.5 T MRI scanner (Siemens Avanto) and the head/neck 8-channel coil were used. The protocol included coronal T2-weighted (w), axial T2w, sagittal T1w, T2w, T2w TIRM (turbo inversion recovery magnitude) sequences, and postcontrast T1w with fat saturation. Only one patient received intravenous contrast. At the atlanto-axial level, bone marrow oedema (BME), effusion, pannus, subluxations, cysts and erosions were looked for (Figure 2). At the subaxial level, BME, apophyseal joint ankylosis, SAS, and vertebral or disc hypoplasia were searched for [10,11]. Anterior AAS was diagnosed when a distance between the posterior aspect of the anterior arch of the atlas and the anterior aspect of the dens exceeded 5 mm [12] (Figure 3).

**Figure 4:**
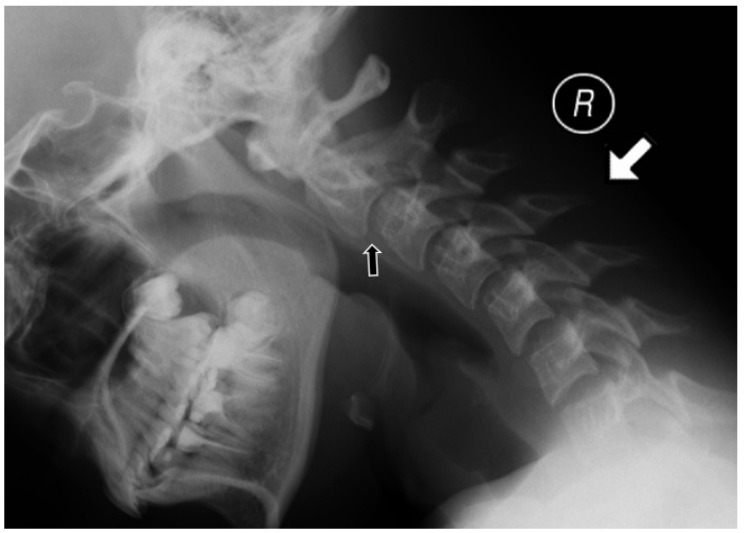
Lateral radiograph in flexion (indicated by white arrow) in a 13-year-old girl showing subaxial subluxation at C2/C3 level (black arrow).

Several methods were used to measure vertical subluxation; the most frequently used methods were McGregor’s (cut off value: 4.5 mm) [13] or the Sakaguchi–Kauppi method. In the latter, vertical AAS is diagnosed when the superior facets of C2 crosses the line formed by the lower aspects of the anterior and posterior arches of C1 vertebra [14]. SAS was reported in patients with the displacement between the upper and lower endplates of adjacent vertebrae exceeding 2 mm (Figure 4) [15].

Since radiography and MRI are not fully compatible in terms of methodology, diagnosed lesions and tissues (functional radiographs in the standing position are superior for the diagnosis of anterior and SAS subluxations, and are more accurate than MRI for the diagnosis of calcifications and ankylosis, whereas MRI in the horizontal position usually does not confirm anterior AAS but it provides evaluation of active inflammation), inflammatory features on radiographs and MRI were assessed separately in a binary way 0: absent, 1 present. Lesions on radiographs were assessed according to study of Espada et al. [9] whereas on MRI they were evaluated on the basis of Hospach et al. and Ključevšek et al. reports [10,11]. Inter-reader reliability was assessed. 

Each radiograph and MRI were evaluated in clinical blinded and randomized order. The data set were evaluated independently by a radiologist with 20 years of experience (ISS) and senior radiology resident (MK; 4 years of experience) both working in rheumatology center. 

In addition, clinical data such as age, sex, disease duration, and current treatment were collected. Laboratory data, including C-reactive protein (CRP; cut-off value 10 mg/L) and erythrocyte sedimentation rate level (ESR; cut-off value 15 mm/h) were extracted, as well as the presence of antinuclear antibodies (ANA; titre higher or equal to 1:160), anticyclic citrullinated peptide antibodies (anti-CPP; cut-off value 17 IU/mL), rheumatoid factor (RF; cut-off value 34 IU/mL), and human leukocyte antigen (HLA) B-27 antigen.

### Statistical Analysis

The SPSS software package (SPSS Inc., Chicago, IL, USA) was used for the purposes of this study. The Shapiro–Wilk test was used to check the normal distribution of continuous variables. Normally distributed data were presented with mean and standard deviation (SD), while non-normally distributed data were presented with median and interquartile range (IQR). Student’s *t*-test was performed for normally distributed continuous data, and a Mann–Whitney U test was used for the analysis of non-normally distributed continuous data. A chi-squared test and Fischer exact test were performed to examine categorical data. *p*-values of less than 0.05 were interpreted as statistically significant. For inter-reader reliability kappa value was used and level of agreement was classified as almost perfect (kappa value above 0.90), strong (0.80–0.90), moderate (0.60–0.79), weak (0.40–0.59), minimal (0.21–0.39), none (0–0.20) [16].

## 3. Results

Thirty-four patients diagnosed with JIA were analyzed (median age 15.5 years; IQR 13.0–17.0 years), of which 25 were girls (74%). The mean age of diagnosis was 9.2 ± 4.5 years, and the mean disease duration was 4.6 ± 3.2 years. In seven children clinically suspected of subluxation dynamic lateral radiographs were performed, while in the remaining 27 patients only radiographs in a neutral position were obtained. 

RF-negative polyarthritis was the most common form of JIA, and it was diagnosed in 13 patients; oligoarthritis was diagnosed in 10 cases, ERA in 5, systemic-onset arthritis in 2, undifferentiated arthritis in 2, and RF-positive polyarthritis and PsA were diagnosed in 1 patient each. 

The cervical spine was affected in 35% (12 out of 34) of the included JIA patients (Table 2). SAS was the most common complication and was found in 8 out of 34 children with JIA (8/34, 24%) and in 2 subjects in non-JIA group (2/38, 5%, *p* = 0.039). All these cases were confirmed by radiography, while only two were seen in MRI. More cases diagnosed with radiography resulted from both the erect positioning of the patient and use of functional projections. 

Three patients (9%) had abnormalities at the C1/C2 level. All had effusion (9%), anterior AAS was seen in 2 patients (6%), vertical AAS in 2 patients (6%), periodontoid pannus in 2 patients (6%) (Figure 2), and BME of the dens in 1 patient (3%). One patient received intravenous contrast and at C1/C2 level enhancement was seen. C1/C2 soft tissue involvement and BME were seen on MRI only. 

Anterior AAS was diagnosed in dynamic radiography (2 cases) but was not confirmed in MRI. Regarding abnormalities that are diagnosed by means of radiography and MRI (Table 3), SAS was seen more commonly on radiographs than MRI.

Three patients (9%) had apophyseal joint ankylosis: two at the C2/C3 level and one at the C3/C4 level, and this was diagnosed by both MRI and radiography in all cases (Table 3). No one developed cysts or erosions of the odontoid process, nor calcification of the anterior or posterior longitudinal ligaments. We did not see any pathology of the brain stem or spinal cord. Regarding subtypes of JIA, most lesions (40%) were seen in patients with ERA, followed by four patients (31%) with RF-negative polyarthritis and three patients (30%) with oligoarthritis. Basilar setting occurred in RF-negative polyarthritis patients only (two cases). Only one patient in the study group had PsA, and no abnormalities in his cervical spine were seen. 

In the whole group, 89% of JIA patients received medical treatment; 18 of them were taking methotrexate, eight steroids, four biological treatments (adalimumab, infliximab, etanercept, or tocilizumab), five chloroquine, and three sulfasalazine. Nine patients were taking two or more drugs. Additionally, 14 patients had positive ANA titer, one had positive anti-CPP values, two had positive RF values, and four patients had the HLA-B27 antigen present. Table 4 shows the differences between patients with cervical spine involvement and those without involvement with regard to analyzed clinical and laboratory data. Cervical spine involvement was associated with a higher concentration of CRP (12.3 versus 6.6 mg/mL; *p* = 0.014) and ESR (24.2 versus 11.9 mm/h; *p* = 0.012). No association was found between cervical spine lesions and disease duration, age at diagnosis, treatment, or peripheral arthritis of the joints.

Interestingly, the only pathology found in non-JIA group was SAS described in 2 children (2/38, 5%). SAS in this group most probably resulted from natural laxity of spine in children described by Lustrin et al. [17].

The overall inter-reader reliability was in most cases almost perfect (kappa value 1.00), despite SAS (0.84) and effusion (0.85), where level of agreement was strong. Regarding anterior AAS (0.79.), ankylosis (0.79) and demineralization (0.66) the observed strength of agreement was moderate (Table 2).

## 4. Discussion

The current study found radiographic and MRI lesions in 35% of JIA patients with clinical suspicion of cervical spine arthritis, including 25% of them developed serious complications, such as atlanto-axial subluxations or ankylosis. Both techniques provided complementary information. 

The most frequent abnormality was SAS. It was seen in eight out of all included patients with JIA (24%), all with 4.5 years history of JIA. All lesions were seen on radiographs, whereas only two were confirmed on MRI which results from the different position of a patient at each examination. In other studies, the prevalence of SAS was lower and affected 6–7% of JIA patients [6,8]. Most commonly, level C4/C5 was affected [8], which was also confirmed in the current study. The lower prevalence of SAS in the mentioned studies could resulted from the coexistence of apophyseal joint ankylosis, which hinders SAS. In this study, SAS was more frequently seen than in other studies with a similar low prevalence of ankylosis. However, this finding was interpreted with caution, bearing in mind that the SAS appearance may result from the natural flexibility of the pediatric cervical spine as seen in 5% of the current non-JIA group and was also described by other authors [17]. 

Ankylosis was seen in 9% of JIA patients. It was diagnosed in patients with late onset disease (mean 12 years of age) however 1.5 year after the diagnosis of JIA. This confirms that ankylosis might occur early in JIA. Espada et al. reported cervical spine ankylosis in 6.5% of patients with JIA during the first year after diagnosis, with 27% occurring during the first five years after diagnosis and the remaining 73% five years or more after the diagnosis. The mean time to develop vertebral fusion was 8.6 years [9]. Other researchers have shown a higher prevalence of cervical spine ankylosis compared with our or Espada et al.’s [3,9,10]. Laiho et al. reported apophyseal joint ankylosis in 41% of patients with JIA, and the mean age of JIA onset was 6.9 years, significantly earlier than in a group without ankylosis [8]. Usually, fusion begins at the C2/C3 level and affects patients with systemic-onset JIA, polyarthritis, or oligoarthritic disease [8,9]. In our group, it developed in two patients with polyarthritis JIA and one with oligoarthritis. In two cases, level C2/C3 was affected. 

Data regarding the occurrence of osseous lesions, such as ankylosis, cysts, erosions, and spinal canal stenosis vary among researchers. Some showed an increased occurrence regardless of the clinical improvement and treatment [10], whereas Ključevšek et al. [11] did not. None of the patient in the current study had cysts or erosions. Only one patient out of 15 studied by Ključevšek et al. developed dens deformation, causing spinal canal compression, thickening of the transverse ligament, and persistent anterior AAS [11]. The lower prevalence of chronic changes could be explained by earlier diagnosis and aggressive treatment with anti-TNF alpha drugs [11]. Several studies have confirmed that successful treatment with biological agents and methotrexate decreases the intensity of inflammation (seen also on MRI as reduction of BME and synovitis) as well as the incidence of complications such as subluxations [10,11].

Some authors suggest that JIA may lead to myelopathy and progressive neurological dysfunction, as in RA; however, there is a lack of publications regarding this topic in JIA [18]. No one from the current study developed lesions in the spinal cord. However, discrete early biochemical changes could not be excluded. A recent study performed by Manczak et al. [19] on adult patients with RA revealed biochemical changes in the spinal cord in patients with anterior AAS diagnosed by apparent diffusion coefficient.

Among our JIA group, 9% had active inflammation at the C1/C2 level (including pannus formation, periodontoid effusions, BME, and postcontrast enhancement). They all occurred within three years from the disease onset. BME was seen in only one patient. In both adults and children, BME is a strong predictor of dens erosions [20]. In our study, nobody developed dens erosions, but in other publications, the prevalence of dens erosions in JIA varied from 13% to 19% [3,6,9]. Hospach et al. in a group of 13 JIA patients, found BME in 93% of the cases; 100% had synovitis and 15% had dens erosions. The high prevalence of all lesions in that study could result from the more common use of MRI in the early stages of the disease in patients with clinical symptoms suggesting cervical spine disease. After treatment with methotrexate and biological therapy, a decrease was noted, with BME seen in 77% and synovitis in 80%. However, despite treatment, the prevalence of dens erosions increased to 31% [10]. 

In the present study, anterior AAS was diagnosed in 6% of cases. Kjellberg et al. [3] reported similar results (5%); however, in young adults with JIA, anterior AAS was found in up to 33% of patients [6]. Basilar setting (vertical AAS) occurred in two of our patients (6%) after 8 years of JIA diagnosis, although in other studies the prevalence of this type of subluxation reaches 13%, and even 25% in young adults with JIA [3,8]. Basilar setting results from severe damage at the C1/C2 level and may lead to brain stem compression or even sudden death [21].

Bone demineralization (bone loss) was seen on radiographs in only one patient with ERA (3% of our group). Typically, it is observed in patients with polyarthritis and systemic-onset disease [22]. Excluding patients treated with steroids, Henderson et al. revealed that low total body bone mineral density is associated with active and severe forms of JIA [23]. Reduced bone mineral density and osteopenia affect young adults following JIA, even those in remission. Both conditions predispose to osteoporosis and bone fractures [24,25].

In the current study only one patient was diagnosed with hypoplasia of the posterior arch of C1 and C2/C3 disc. This is entirely different from data presented by other authors, where vertebral and disc hypoplasia are regarded as the second most common complication in JIA. They are observed in patients with earlier onset of JIA and are almost always seen at the level of ankylosis [9]. We can presume that the prevalence of vertebral hypoplasia and other chronic lesions is less common nowadays in the era of biological treatment. Vertebral and disc hypoplasia were also associated with a severe course and progression of JIA. Indeed, in the present study, the patient with vertebral hypoplasia of the C1 posterior arch and C2/C3 disc hypoplasia developed ankylosis, SAS, and vertical AAS. Most frequently, hypoplasia is seen at the C3–C6 level, and it is probably caused by inflammation or/and aggressive pharmacotherapy with steroids [26]. The fourth cervical vertebra is most severely affected by hypoplasia (up to 26% of cases), whereas the spinal canal diameter is almost the same as in the healthy population [6,8,26].

No cases of anterior or posterior longitudinal ligament calcification were observed in the present study. Espada et al. [9], in their study published in 1988, reported posterior longitudinal ligament calcification in 6.6% of the patients, and almost half of these developed anterior longitudinal ligament calcifications. The disease duration was approximately 13 years prior to the onset of calcification, and a slightly higher occurrence was noted in males and in individuals with earlier disease start (3.8 years). All patients with longitudinal ligament calcification had multilevel ankylosis, suggesting that chronic immobilization due to ankylosis could lead to ligamental calcification [9]. This study again confirms the more successful treatment of JIA nowadays. 

In the current study, cervical spine involvement was associated with elevated ESR (*p* = 0.012) and CRP (*p* = 0.014). According to the ACR, CRP and ESR are considered biomarkers of JIA activity [4], and their prolonged elevation is associated with poor prognosis in JIA [4] and with lack of remission [27]. Additionally, ESR seems to predict the development of uveitis in JIA [28]. Although CRP and ESR have low specificity and sensitivity, they were found useful in the diagnosis and follow-up of patients with JIA. ESR was also included in the Juvenile Arthritis Disease Activity Score (JADAS) [29]. 

Overall, although MRI may show early inflammatory lesions, the dynamic radiography is superior in the diagnosis of cervical spine instabilities (Table 3). Both severe AAS and SAS may cause cervical cord pathologies, even including sudden death. Thus, functional radiography should be still performed in the suspicion of cervical spine instabilities. The radiography is cheap and widely available, but the major disadvantages include radiation, superimposition of anatomical structures and limited use for soft tissues imaging. In comparison, MRI is superior in assessment of soft tissue pathologies, spinal cord and nerve roots, and may visualize BME. However, MRI is expensive and due to long time of acquisition young children or claustrophobic patients may require sedation. 

The major limitation of the study is the retrospective study protocol. The small number of JIA group (34 and cervical spine lesions in 12) does not allow making robust comments about the results of the study. Another limitation resulting from the nature of JIA underlined in literature is a lack of direct correspondence between cervical pain and presence of alterations on radiographs and MRI since silent cervical spine arthritis has also been reported [5]. The current study focused on patients previously diagnosed with JIA and clinical symptoms suggesting cervical spine arthritis. Only patients examined with two imaging methods were enrolled and no asymptomatic children with JIA were analyzed. It would have been better to extend the investigation including also JIA patients with no cervical symptoms in order to evaluate the eventual presence of silent alterations in this group. Whereas from an ethical point of view it is impossible for radiography, it is possible for MRI. Finally, the major strength and originality of our study was the presentation of both radiographic and MRI features of cervical spine involvement in JIA. Previous studies focused only on one method.

## 5. Conclusions

In conclusion, cervical spine lesions may affect up to 35% of JIA patients, and 25% of them develop serious complications, such as atlanto-axial subluxations and ankylosis. Despite clear advantages of MRI in terms of imaging of early inflammatory lesions in soft tissues and bone, radiography shows superiority in the diagnosis of AAS and SAS. The predominance of chronic features, such as SAS, AAS and ankylosis over early inflammatory abnormalities, including BME and synovitis along with the several years of history of JIA suggest that clinical manifestation of cervical spine involvement is discrete or even absent in the first years of the disease, which lets the cervical arthritis progress unrecognized until a more advanced stage.

## Figures and Tables

**Table 1:** Comparison between JIA and non-JIA patients.

	JIA Group	Non-JIA Group	*p*
Number	34	38	
Age (years) *	15.5 (13.0–17.0)	15.0 (13.0–16.0)	0.668
Sex (%)	Female: 25 (74%) Male: 9 (26%)	Female: 28 (74%) Male: 10 (26%)	1.000

JIA—juvenile idiopathic arthritis, * non-normally disturbed data: median and interquartile range were used.

**Table 2:** Prevalence of different lesions in cervical spine in patients diagnosed with JIA.

Kerrypnx		RF Negative Polyarthritis (*n* = 13)	RF Positive Polyarthritis (*n* = 1)	Oligoarthritis (*n* = 10)	ERA (*n* = 5)	Systemic-onset Arthritis (*n* = 2)	uA (*n* = 2)	PsA (*n* = 1)	Total (*n* = 34)	Control Group (*n* = 38)	Interobserver Kappa Value
Prevalence in JIA		11–28%	2–7%	27–56%	3–11%	4–17%	11–21%	2–11%			
Study prevalence *		38%	3%	29%	15%	6%	6%	3%			
C1/C2 level	BME	–	–	–	1	–	–	–	1 (3%)	0 (0%)	1.0
	Effusion	2	–	–	1	–	–	–	3 (9%)	0 (0%)	0.85
	Pannus	1	–	–	1	–	–	–	2 (6%)	0 (0%)	1.0
	Contrast enhancement **	–	–	–	1	–	–	–	1 (3%)	0 (0%)	1.0
	Dens erosions	–	–	–	–	–	–	–	0 (0%)	0 (0%)	1.0
	Anterior AAS	1	–	–	1	–	–	–	2 (6%)	0 (0%)	0.79
	Vertical AAS	2	–	–	–	–	–	–	2 (6%)	0 (0%)	1.0
SAS		2	1	2	1	1	1	–	8 (24%)	2 (5%)	0.84
Ankylosis		1	1	1	–	–	–	–	3 (9%)	0 (0%)	0.79
hypoplasia of vertebral body or disc		1	–	–	–	–	–	–	1 (3%)	0 (0%)	1.0
Demineralization		–	–	–	1	–	–	–	1 (3%)	0 (0%)	0.66
Total		4 (31%)	1 (100%)	3 (30%)	2 (40%)	1 (50%)	1 (50%)	0 (0%)	12 (35%)	2 (5%)	

JIA—juvenile idiopathic arthritis, ERA—enthesitis-related arthritis, PsA—psoriatic arthritis, uA—undifferentiated arthritis, RF—rheumatoid factor, BME—bone marrow edema, AAS—atlanto-axial subluxation, SAS—subaxial subluxation, * visualized by at least one technique, ** contrast was given in 1 case only.

**Table 3:** Number of patients with JIA (total *n* = 34) with lesions diagnosed on radiography and MRI.

Pathology	Radiography	MRI
Anterior AAS	2	0
Vertical AAS	2	2
SAS	8	2
Dens erosions	0	0
Ankylosis	3	3
Hypoplasia of vertebral body or disc	1	1

All differences were not statistically significant (*p* > 0.05). MRI—magnetic resonance imaging, AAS—atlanto-axial subluxation, SAS—subaxial subluxation.

**Table 4:** Comparison between JIA patients diagnosed with cervical spine involvement on imaging with JIA patients without confirmed cervical spine involvement.

Kerrypnx		Cervical Spine Involvement (*n* = 12)	No Cervical Spine Involvement (*n* = 22)	*p*
Age (years) *		15.0 (13.0–16.8)	15.5 (12.3–17.0)	0.817
Sex (female, %)		10 (83%)	15 (68%)	0.439
Age at onset (years)		9.9 ± 4.1	8.5 ± 4.7	0.604
Disease duration (years)		4.4 ± 2.8	4.7 ± 3.4	0.813
CRP (mg/mL) *		8.0 (5.0–14.5)	4.0 (2.5–5.5)	0.014
ESR (mm/h) *		29.5 (11.5–39.0)	9.0 (5.0–17.5)	0.012
ANA positivity (*n*,%)		8 (80%)	10 (59%)	0.406
Treatment	Methotrexate (*n*,%)	8 (80%)	10 (59%)	0.406
	Steroids (*n*,%)	5 (50%)	3 (18%)	0.102
	Chloroquine	1 (10%)	4 (24%)	0.621
	Biological treatment (*n*,%)	3 (30%)	1 (6%)	0.128

JIA—juvenile idiopathic arthritis, CRP—C-reactive protein, ESR—erythrocyte sedimentation rate, ANA—antinuclear antibodies, * non-normally disturbed data: median and interquartile range were used. The bold means significant.

## Data Availability

The study data may be available on request. The data presented in this study are available on request from the corresponding author. The data are not publicly available due to ethical concerns.
